# Retinal Vessel Coronal Displacement in Intermediate Age-Related Macular Degeneration

**DOI:** 10.3390/jcm14176030

**Published:** 2025-08-26

**Authors:** Mariacristina Parravano, Serena Fragiotta, Maria Sole Polito, Monica Varano, Giorgio Querzoli, Tommaso Rossi

**Affiliations:** 1 IRCCS Fondazione Bietti ONLUS, 00198 Rome, Italy; mariasole.polito@fondazionebietti.it (M.S.P.); monica.varano@fondazionebietti.it (M.V.); tommaso.rossi@fondazionebietti.it (T.R.); 2 Faculty of Medicine, UniCamillus-Saint Camillus International University of Health Sciences, 00131 Rome, Italy; 3 Ophthalmology Unit, NESMOS Department, St. Andrea Hospital, Sapienza University of Rome, 00189 Rome, Italy; serena.fragiotta@uniroma1.it; 4 Department of Civil and Environmental Engineering and Architecture (DICAAR), Università di Cagliari, 09123 Cagliari, Italy; querzoli@unica.it

**Keywords:** optical coherence tomography angiography, microvascular retinal plexuses, age-related macular degeneration

## Abstract

**Introduction**: This pilot study aimed to test the feasibility of a novel vectorial image analysis method to quantify coronal microvascular displacement in different retinal plexuses in intermediate age-related macular degeneration (iAMD) over 6 months. **Material and methods**: A retrospective series of iAMD patients with at least 6-month follow-up was included if they had complete medical records, best-corrected visual acuity (BCVA), optical coherence tomography (OCT), and OCT angiography (OCTA). En-face (coronal) vascular displacement between baseline and 6 months was assessed in the superficial capillary plexus (SCP), deep capillary plexus (DCP), and choriocapillaris (CC) using the Farneback motion tracking algorithm applied to consecutive OCTA scans. **Results:** Eighteen eyes of 18 iAMD patients met the inclusion criteria. Average coronal vascular displacement (T_0_–T_6_) was 13.7 ± 7.72 µm for the SCP, 15.11 ± 10.06 µm for the DCP, and 19.02 ± 12.25 µm for the CC slab. Reticular pseudodrusen (RPD) was associated with greater displacement in the DCP (*p* = 0.047), but not in the SCP (*p* = 0.980) or CC (*p* = 0.473). Quantitative analysis confirmed the highest DCP displacement in RPD eyes (66.7%, *p* = 0.02), while drusenoid pigment epithelial detachment showed the greatest reorganization in the CC (100%, *p* = 0.02). **Discussion:** Retinal vessels undergo significant tangential displacement in iAMD, suggesting a structural reorganization of the microvasculature. Such remodeling may constitute a compensatory response to ultrastructural alterations resulting in ischemia.

## 1. Introduction

Age-related macular degeneration (AMD) is the leading cause of legal blindness in developed countries. It has a multifactorial pathogenesis, influenced by aging, environmental risk factors, and genetic susceptibility [1]. Choriocapillaris dropout is part of the aging process and becomes evident from the early stages of AMD, preceding the development of late-stage complications [2,3,4]. The intimate relationship among photoreceptors, retinal pigment epithelium (RPE) and its basal lamina, extracellular deposits, and choriocapillaris in the pathogenesis of AMD and its complications has been extensively discussed [2,5]. A reduction in vascular density has also been reported at the level of the superficial and deep vascular plexi through optical coherence tomography angiography (OCTA), along with the involvement of the inner retina. The exact pathological relationship between the retinal and microvascular changes remains to be elucidated, but the loss of vascular density is likely part of the vascular remodeling observed in AMD [6,7].

Vascular endothelial growth factor (VEGF) has been considered the most important regulator of angiogenesis in AMD, expressed under hypoxic conditions [8,9]. VEGFA and VEGFB are both involved in macular neovascularization (MNV), participating in vessel formation and growth, vascular permeability, support, and remodeling of the neovascular membrane [10]. Other factors such as platelet-derived growth factor (PDGF) are involved in vascular remodeling, characterized by activation and remodeling of the extracellular matrix and smooth muscle cells of the vascular wall. This process, known as arteriogenesis, mediates vascular dilatation of pre-existing vessels under the influence of shear stress without capillary proliferation [11,12,13]. In a macaque model, oxygen redistribution has been demonstrated to preserve photoreceptors from hypoxia, with the retinal circulation contributing approximately 10–15% of the total oxygen supply [14].

Profound vascular remodeling throughout the course of the disease has been described in all AMD forms, as the two variants represent the extremities of one continuous spectrum of disease in which one form can progress to another [15,16]. Intense vascular remodeling is a hallmark of disease progression and has been documented in the choroid [17]. This remodeling includes vessel dilation, branching patterns, neovascular sprouting, and redistribution of perfusion across the retinal and choroidal layers, as seen in both experimental models and clinical imaging studies [14,15,17,18,19]. Despite clear evidence of vascular density modifications in AMD, their clinical and prognostic significance remains uncertain. In this context, such microvascular changes may also be interpreted as a compensatory adaptation driven by hypoxia-induced molecular signaling and shear stress.

We recently introduced a novel image analysis method that calculates the vector displacement of retinal structures, including epiretinal membrane and retinal microvasculature [20,21]. However, in these studies, vascular displacement was assessed on infrared images, where vectors may belong to different vascular plexuses, such as superficial and deep. More recently, the same methodology was applied to consecutive OCTA images, allowing for tracing the coronal vascular displacement across the different retinal vascular plexuses [22]. This method applied to eyes with intermediate AMD (iAMD) may help to clarify the role of vascular remodeling as both a protective and pathogenic mechanism driving late-stage complications. In fact, although OCTA technology is widely used in the clinical setting, it primarily provides information on blood flow within retinal vessels, without assessing how the structural organization of these vessels may change in response to retinal hypoxic stress [23,24]. Previous studies have demonstrated that oxygen tension is reduced by 30–50% over drusen and large drusenoid pigment epithelial detachments (dPED). Such perfusion changes can trigger intraretinal migration of RPE cells, fostering a cytokine environment conducive to vascular remodeling in the deep capillary plexus (DCP) [25,26,27].

Given the absence of prior evidence-based studies on coronal vascular displacement in iAMD, this pilot investigation was designed to generate preliminary data and assess the in vivo microvascular remodeling. Furthermore, we hypothesized that microvascular displacement patterns would differ among retinal and choroidal plexuses according to specific iAMD phenotypes.

## 2. Materials and Methods

### 2.1. Study Participants

This retrospective cohort analyzed vascular displacement between 2 consecutive follow-up visits (baseline and after 6 months) in 18 patients (18 eyes) diagnosed with iAMD. All patients provided written informed consent; the study adhered to the tenets of the Declaration of Helsinki and received approval from the local Ethics Committee (N.114/21/FB, approval date: 26 January 2021).

Subjects were followed at the IRCCS Bietti Foundation between June 2018 and November 2018. The diagnosis of iAMD was based on ophthalmoscopic examination according to the Beckman classification [28] and confirmed using fundus autofluorescence and spectral-domain optical coherence tomography (SD-OCT).

Inclusion criteria were the availability of complete medical records and multimodal imaging to ensure comprehensive clinical and imaging assessment, including SD-OCT with simultaneous acquisition of near-infrared reflectance (NIR), OCTA, and infrared retinography. Exclusion criteria were applied to reduce confounding factors and avoid artifacts that could bias the analysis, as follows: (1) history of ophthalmological disorders, particularly retinal disorders other than AMD, including degenerative, traumatic, vascular, and inherited retinal disorders, high myopia (axial length > 26 mm), and uveitis; (2) history of systemic disorders, including arterial hypertension and diabetes mellitus; (3) poor imaging quality due to media opacity or image artifacts; (4) incomplete medical records.

Conventional drusen were diagnosed as multiple RPE elevations with variable internal reflectivity on OCT B-scans. Reticular pseudodrusen (RPD), also known as subretinal drusenoid deposits (SDDs), were recognized as conical or flat hyperreflective deposits within the RPE and the boundary between the inner and outer segments of photoreceptors, better seen on infrared reflectance. The presence of dPED was diagnosed through clinical and multimodal imaging evaluations, adopting the Age-Related Eye Disease Study criteria, with a minimum horizontal diameter of 350 μm [29].

### 2.2. Optical Coherence Tomography Image Acquisition

OCTA images were obtained using the ANGIOVUE software integrated with the commercially available RTVue XR spectral domain-OCT device (Optovue, Inc., Fremont, CA, USA). This instrument features an A-scan rate of 70,000 scans per second, using a light source centered at 840 nm with a bandwidth of 45 nm. A split-spectrum amplitude-decorrelation angiography (SSADA) algorithm, explained in detail elsewhere [23], was used to detect erythrocyte movement and depict blood flow in a 3 × 3 mm^2^ scanning area centered on the fovea.

Enface OCTA angiograms were segmented using the built-in software segmentation algorithm to define the superficial capillary plexus (SCP), DCP, and choriocapillaris (CC). The 3 × 3 mm^2^ volumetric images were processed using the 3D projection artifact removal (PAR) tool provided by ANGIOVUE software.

Image quality was ascertained by excluding images with a quality index of <5 and/or motion artifacts. Motion artifacts or poor image quality were revised by a single expert operator (T.R.) and excluded prior to the analysis.

### 2.3. Displacement Measures

Dislocation was evaluated by measuring the displacement between pairs of consecutive 3 × 3 mm^2^ wide images acquired through RTVue XR spectral domain-OCT device (Optovue, Inc., Fremont, CA, USA) by means of an optical flow algorithm described in detail elsewhere^6^. The microvascular displacement during the first 6 months was assessed by comparing baseline and 6-month follow-up (T_0_–T_6_).

Displacements between consecutive OCTA slabs were computed on a regular grid composed of 36 × 36 nodes. To ensure precise and reproducible quantification of vascular displacement, the image processing consisted of the following steps: image pre-processing, alignment, motion estimation, and post-processing [20,21,22]. All OCTA images were first converted to grayscale and subjected to local histogram equalization to standardize contrast. Rigid roto-translational alignment was then applied to correct image orientation and centration, ensuring precise alignment, as previously detailed [21]. In brief, a two-component displacement map was computed using the Farneback algorithm. Next, the roto-translation that minimized the sum of squared differences across the 36 × 36 displacement vectors was determined and applied to the second image. This alignment step was iteratively repeated until convergence, defined as a change of less than 3% in the sum of squared differences between consecutive iterations. After alignment, microvascular displacements were measured using the Farneback two-frame motion estimation algorithm [30], which estimates a dense displacement field by comparing the neighborhoods of corresponding nodes as quadratic polynomial expansions and tracking their translation between images. The resulting per-pixel displacement vectors (dx, dy) were expressed in micrometers, and the displacement magnitude for each vector was computed as $D=\sqrt{Dx^{2}+Dy^{2}}\;$ [22].

**Figure 1:**
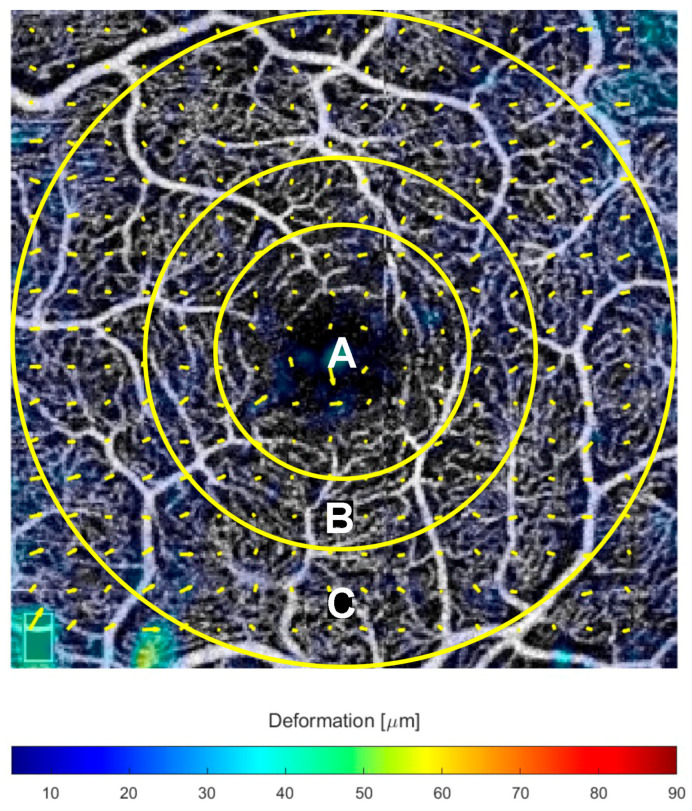
Optical coherence tomography angiography (OCTA) of the superficial capillary plexus, with the overlaid regions used to analyze the retinal deformation: (A) central circle with a radius of 0.5 mm; (B) the second concentric circle has a radius of 0.75 mm; (C) the outermost circle with a radius of 1.5 mm.

Measurements were obtained for predefined regions of interest: (i) whole image; and (ii) three circular regions centered on the foveola with radii equal to 0.5 mm, 0.75 mm, and 1.5 mm (Figure 1). The 0.5 mm radius corresponds to the central subfield (1 mm diameter) and encompasses the foveal avascular zone (FAZ); the 0.75 mm radius (1.5 mm) lies between the central subfield and part of the parafovea; and the 1.5 mm radius (3 mm) corresponds to the parafovea. This multiscale sampling enables the detection of spatially dependent patterns of microvascular displacement.

The final output consisted of vascular slabs overlaid with yellow arrows indicating vectorial displacement direction between baseline and 6 months, with arrow length representing the absolute value of the vector. The magnitude of the vascular displacement is expressed as deformation with a color-coding (Figure 1, bottom), where red signifies the greatest displacement, measured in micrometer (μm). The deformation scale is comprised between 0 and 90 μm.

### 2.4. Statistical Analysis

Quantitative data are expressed as mean ± standard deviation together with 95% confidence intervals (95% CI), unless otherwise specified. Given the pilot nature of the study, a formal sample size calculation was not conducted; the sample size was determined by the number of eligible cases meeting the inclusion criteria within the study period. The Shapiro–Wilk test was employed to evaluate the normality of the data distribution. A paired t-test was performed to assess changes at the different time points (T_0_ and T_6_). Cohen’s d was calculated to estimate effect sizes. To compare vascular displacement among different microvascular slabs, a linear mixed-effects model was applied with slab type (CC, DCP, SCP) as a fixed effect and patient ID as a random effect to account for repeated measures. The CC slab was used as the reference category. Model assumptions (normality of residuals, homoscedasticity) were verified prior to inference. Estimated marginal means with 95% CI were reported, along with effect sizes and *p*-values for fixed-effect contrasts. All statistical tests were two-tailed, and *p*-values < 0.05 were considered statistically significant.

## 3. Results

Eighteen eyes of 18 patients with iAMD (8 males and 10 females) were included in the study. The mean age was 68.1 ± 7.9 years. The main characteristics of the study subjects are reported in Table 1.

### 3.1. En-Face Retinal Displacement

Average coronal vascular displacement calculated between baseline and 6-month follow-up (T_0_–T_6_) was 13.69 ± 8.65 µm for the SCP, 15.10 ± 12.72 µm for the DCP, and 19.02 µ ± 12.67 µm for the CC slab. Average displacement values at different eccentricities are presented in Table 2.

Different degrees of coronal vascular displacement were observed at all microvascular levels in iAMD eyes. The linear mixed model confirmed a significant difference in displacement among layers (*p* < 0.01), with estimated marginal means reported in Table 3.

Patients showing RPD presented a significantly higher vascular displacement in the DCP (*p* = 0.04), but neither in the SCP (*p* = 0.98) nor in the CC (*p* = 0.47).

### 3.2. Qualitative Features Associated with a Tangential Vascular Displacement

**Figure 2:**
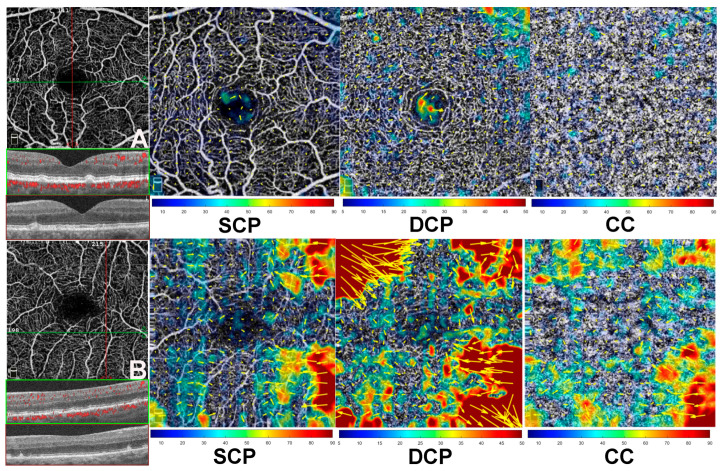
Examples of vascular displacement in a case with drusen compared to a case with reticular pseudodrusen (RPD). (**A**), Case with drusen: the original superficial capillary plexus (SCP) slab is shown alongside the corresponding horizontal (green) and vertical (dark red) B-scans. Microvascular layers are displayed as SCP, deep capillary plexus (DCP), and choriocapillaris (CC). The output displays yellow arrows indicating the vectorial displacement direction, with arrow length proportional to the displacement magnitude. The deformation is color-coded (bottom), where red represents the greatest displacement, measured in micrometers. Displacement vectors represent the vascular dislocation observed between baseline visit and 6-month follow-up. (**B**), case with RPD: demonstrates more pronounced deformation, with vectors pointing centripetally, in contrast to the case with drusen (**A**), where only subtle changes are evident. Vascular displacement is most pronounced at the DCP, where an impressive remodeling is evident as vectors (yellow arrows) and deformation expressed in microns (dark red). The colorimetric scale displayed at the bottom of the figure provides a legend of the deformation measurements expressed in microns (μm). In contrast, the case with drusen ((**A**), DCP) shows only minimal changes, primarily confined to the central region. At the CC, subtle changes are observed in the case with drusen, mostly in the outermost superior region. However, the case with RPD demonstrates significant changes in the displacement vectors, indicating notable vascular remodeling.

**Figure 3:**
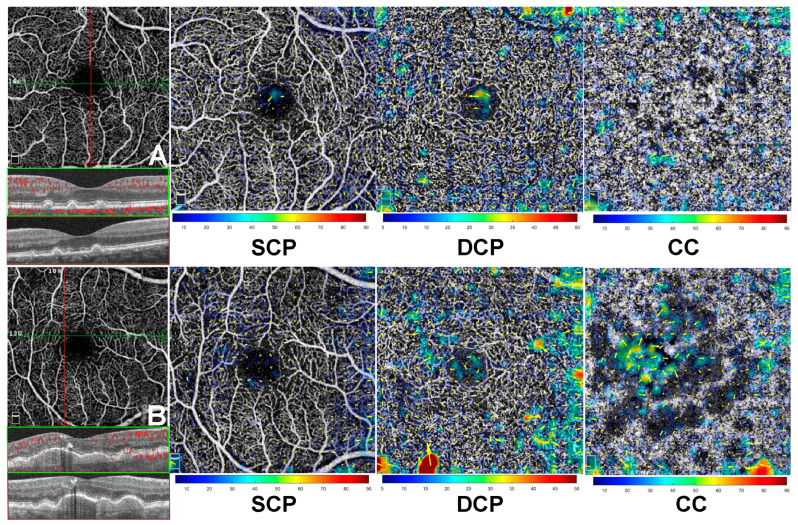
Examples of vascular displacement in a case with drusen compared to drusenoid pigment epithelial detachment (dPED). Displacement vectors represent the vascular dislocation observed between baseline visit and 6-month follow-up. (**A**), case with drusen and (**B**) case with dPED, both demonstrating no relevant changes in the vascular displacement at superficial capillary plexus (SCP). At deep capillary plexus (DCP), the displacement vectors are more represented, particularly in the case with dPED (**B**) when compared to drusen alone (**A**). At choriocapillaris (CC), subtle changes are observed in the case with drusen ((**A**), CC panel), mostly in the outermost superior region. By contrast, the case with dPED ((**B**), CC panel) demonstrates changes in the displacement vectors, particularly in the central and outermost regions (inferior, nasal, and temporal).

Eyes with the greatest vascular displacement in the DCP showed a significant association with RPD in 6/9 eyes (66.7%, *p* = 0.02), while the remaining 3/9 eyes (33.3%) developed iRORA after 6 months of follow-up. An exemplary case is shown in Figure 2. Moreover, eyes with the greatest vascular displacement observed at the CC level demonstrated dPED at 6 months of follow-up in all the cases considered (3/3 eyes, *p* = 0.02). Among eyes with similarly mild vascular displacement changes between DCP and CC, the preponderant phenotype was characterized by conventional drusen in 5/6 eyes (83.3%) with a tendency toward confluence over the 6-month follow-up (*p* = 0.68). For further details, see Figure 3.

## 4. Discussion

The present study investigates the microvascular coronal displacement in iAMD eyes over a 6-month follow-up. Retinal vascular displacement was calculated using a recently developed image analysis algorithm applied to consecutive OCTA slabs [20,21]. This method estimates displacement by comparing pairs of images acquired at different time points to detect motion between them [30]. The use of OCTA images allows the calculation of coronal displacement across different microvascular networks. The main advantage of this approach is its ability to provide separate, layer-specific analyses of each microvascular plexus [24].

By examining different retinal vascular plexuses, this study observed microvascular remodeling at all levels, particularly evident in the DCP and CC. Extracellular deposits characteristic of AMD such as basal laminar deposits (BLamDs) and basal linear deposits, drusen, dPED, and RPD are thought to act as a physical barrier to retinal oxygenation [25,31,32,33,34]. Such choroidal hypoxia may contribute to intraretinal migration of RPE cells, inducing a cytokine environment that supports vascular reorganization in the DCP, with or without neovascular potential [25]. Microvascular anomalies at the DCP level have been described in the absence of neovascularization [27]. Neovascular complexes originating at this level may be promoted by inflammation and ischemia, a substrate that could also contribute to vascular remodeling. In this context, microvascular modifications might facilitate blood flow to a newly growing neovascular complex, involving both newly formed or pre-existing vessels [35,36]. Therefore, although reduced vessel density in DCP is often more evident in advanced disease stages [37,38], the ability of our method to detect microvascular displacement patterns offers a novel means of capturing early microvascular adaptations that may precede clinically evident atrophy or neovascularization.

Interestingly, the retinal microvascular displacement varied among microvascular plexi according to the phenotypic iAMD lesions. In this regard, RPD was associated with the greatest displacement at the DCP level, and eyes with the most relevant changes in the DCP were predominantly associated with RPD (66.7%). Few studies have explored the relationship between the SCP and DCP microvascular parameters in the setting of RPD. A reduced DCP vessel density has been reported in eyes with RPD, which has been linked to inner retinal thinning [7,39]. However, most of the existing literature reports significant ischemic changes at the level of CC [40,41]. Therefore, it has been proposed that RPD may originate from pre-existing localized choroidal hypoperfusion, which could be exacerbated following RPD formation [42]. By analyzing topographical vessel perfusion on OCTA slab, a recent study by Nam et al. [43] revealed decreased vessel perfusion on both SCP and DCP, extending nasally and superiorly from the fovea. The authors speculated a direct role of RPD in vascular impairment based on the observed spatial co-localization between RPD and microvascular alterations. Moreover, the involvement of the inner retina in RPD reported in several studies [7,39,44] has opened the hypothesis of a trans-synaptic degeneration driven by suffering photoreceptors or a consequence of chronic and progressive hypoperfusion [7,44]. Notably, a recent experimental study has tested the possible role of RPE hypoxia, secondary to CC hypoperfusion, in the development of RPD/SDD. Under normoxic conditions in AMD, apoE-containing lipopoprotein particles are secreted both apically into the subretinal space and basally into the sub-RPE space, contributing to the formation of RPD/SDD and drusen, respectively. However, under severe hypoxic stress, RPE polarity becomes disrupted, resulting in preferential apical secretion of apoE and reduced basal secretion. This imbalance may exacerbate pre-existing choroidal hypoperfusion, observed in eyes with prominent RPD/SDD but few drusen, accompanied by photoreceptor outer segment abnormalities [45,46]. This mechanism may hypothetically help explain why the most prominent vascular remodeling occurs in the deep retinal plexus rather than the CC, which is already compromised. Our finding of greater displacement in the DCP may represent a secondary, compensatory remodeling process. In this context, DCP reorganization is consistent with the hypothesis of an adaptive response aimed at maintaining photoreceptor oxygenation when CC is hypoxic. This hypothesis complements, rather than contradicts, the established role of CC impairment in RPD pathophysiology. Whereas OCTA primarily assesses vessel flow, our innovative method measures microvascular displacement, representing the spatial shift in the vascular network observed between consecutive follow-ups. This measure is independent of flow detection and instead reflects the coronal displacement of the microvasculature, indicating a reorganization of vascular arborization in response to hypoxic conditions.

It has been hypothesized that part of the apparent loss in vascular density seen in AMD may actually result from vascular remodeling, which involves alterations in vessel length, diameter, branching patterns, and overall complexity [47]. An experimental animal model of AMD demonstrated that microvascular changes related to hypoxia can precede neovascularization [19]. These changes include vascular congestion, vesiculation, increased endothelial thickness, luminal projections from endothelial cells, and splitting existing extravascular columns. Before neovascularization, vascular leakage may involve the dissolution of tight junctions and the enlargement of endothelial fenestrations [19]. These ischemic changes in the choriocapillaris, observed in eyes with RPD, could contribute to a reorganization of vascular plexuses, leading to remodeling of the DCP. This vascular plexus has been proven to contribute, on average, 15% to photoreceptor oxygenation under dark adaptation and 11% in light conditions, increasing to 23% under both conditions at the edge of the avascular region [14].

The choriocapillaris dropout in AMD is well documented [3,16,48,49], and choriocapillaris vulnerability increases in iAMD compared to physiological aging and early AMD [50]. However, imaging CC flow with OCTA has several limitations. These include signal attenuation beneath drusen or dPED, choroidal hypertransmission caused by atrophy, and segmentation inaccuracies, all of which can lead to misinterpretation when evaluating CC flow [51,52]. Our approach, therefore, offers a different perspective on microvascular reorganization when flow is compromised. Notably, our study observed that the greatest changes in CC vascular displacement were detected in eyes with dPED, a phenotype often associated with marked alterations in choroidal perfusion. In dPED, the structural modifications of the RPE are thought to be proportional to the volumetric changes in the PED [53]. The legacy of RPE alterations accompanying dPED can result from intrinsic signaling to the RPE cells, inducing apoptosis and migration, or as a consequence of prolonged ischemia [53,54]. In this regard, the increased distance between the choriocapillaris-Bruch’s membrane from RPE-BL and the outer retina can create chronic and progressive ischemia, leading to RPE and photoreceptor dysfunction [54]. A progressive choroidal thinning during the dPED lifecycle may further reinforce a possible impairment of choriocapillaris and choroidal vasculature [55]. Areas of RPE loss have been reported to co-localize with a reduced choriocapillaris perfusion, characterized by severe vasoconstriction and loss of fenestrations [56]. Viable RPE cells produce VEGF, which may stimulate the formation of fenestrations and vasodilatation of choroidal circulation [9]. Therefore, it is plausible that, since most RPE alterations occur at the PED apex –where the separation from the underlying choriocapillaris is greatest—the residual RPE cells increase the production of VEGF and other growth factors in an attempt to improve the choriocapillaris microcirculation [57,58]. The resulting microvascular reorganization may be detectable through our vectorial displacement model, which focuses on remodeling of residual capillaries rather than only on flow deficits [4,48].

This study has several limitations, including a small sample size, a relatively short longitudinal follow-up, and a focus on the direct correlation between microvascular perfusion parameters. The small sample size (18 eyes) limits statistical power and generalizability, especially given the phenotypic heterogeneity of iAMD. Furthermore, potential confounding factors such as high axial length, systemic vascular conditions, and segmentation artifacts were minimized through strict exclusion criteria. As a pilot study, the primary aim was to assess the feasibility and clinical relevance of a novel vectorial image analysis method. Correlations with functional outcomes such as BCVA or microperimetry were not performed because microperimetry data were largely unavailable, and the limited sample would have yielded underpowered analyses. However, the innovative approach used in this analysis provides preliminary insights into microvascular reorganization in AMD that appear to vary according to the different phenotypic lesions observed. Future studies with larger, prospectively enrolled cohorts, extended follow-up, and a broader range of phenotypic presentations are needed to validate these preliminary observations, refine the measurement methodology, and assess the prognostic utility of microvascular displacement as a biomarker in iAMD. Despite these limitations, the consistent trends observed across phenotypes support the biological plausibility of the findings and provide a rationale for further investigation. The microvascular rearrangement was more prominent at the DCP level. In this regard, RPD and the development of outer retinal atrophy were associated with the most significant changes observed in the DCP. Meanwhile, microvascular displacement in the choriocapillaris was most frequently associated with the development of dPED, a finding that may be influenced by significant ischemia due to the increased separation between the outer retina and RPE.

## 5. Conclusions

This study demonstrates that microvascular plexuses in iAMD undergo distinct patterns of coronal displacement over time, suggesting active remodeling processes. The degree and distribution of vascular displacement differ across plexuses and are influenced by specific phenotypic lesions such as RPD and dPED, reflecting underlying pathophysiologic mechanisms. In eyes with RPD, the greatest remodeling was observed within the deep retinal circulation, consistent with the hypothesis of microvascular adaptation to early and diffuse CC ischemia aimed at maintaining photoreceptor oxygenation. In dPED, the separation between photoreceptors and the RPE is greatest at the lesion apex, creating a potential niche where the surrounding choriocapillaris may contribute to the delivery of oxygen and nutrients.

From a translational perspective, the ability to detect early microvascular displacement patterns in iAMD phenotypes may help clarify the pathogenic model of vascular adaptation to hypoxic stress. Our findings suggest that specific phenotypes, such as RPD and dPED, exhibit distinct remodeling responses in the DCP and CC, respectively, which may reflect different adaptive strategies to maintain retinal metabolic demands. Understanding these early adaptive mechanisms could refine current disease models and provide a framework for interpreting vascular changes observed with OCTA. While the present pilot study was not designed to directly inform clinical guidelines, and its inherent design limitations must be acknowledged, it provides preliminary evidence that may help bridge structural imaging biomarkers with the underlying pathophysiological processes in AMD. Moreover, understanding these microvascular dynamics may help predict the pathogenic cascade leading to macular complications and support the development of more targeted monitoring and therapeutic strategies. Future prospective, longitudinal studies with larger cohorts are warranted to validate these findings, explore their prognostic value, and further elucidate the role of microvascular displacement in iAMD.

## Figures and Tables

**Table 1:** Main characteristics of the intermediated age-related macular degeneration (iAMD) patients enrolled at baseline (T_0_) and 6 months of follow-up (T_6_).

Time Points				
Variables	T_0_	T_6_	CI 95%	*p* (T_0_–T_6_)
BCVA (ETDRS letters)	82.17 ± 4.54	81.78 ± 5	−3.59, 2.81	0.80
Snellen equivalent	20/25	20/25	-	-
Macular Thickness (µm)	287.8 ± 32.3	287.9 ± 33.4	−16.49, 16.69	0.99

BCVA: Best corrected visual acuity; ETDRS: Early Treatment Study Group; CI 95%: 95% confidence interval for the paired mean difference. Values are expressed as mean ± standard deviation. Snellen equivalent is expressed as a fraction, representing the conversion of BCVA.

**Table 2:** Average tangential vascular displacement in microns (T_0_–T_6_) calculated over the entire observation period at different eccentricities.

	Vascular Displacement (µm)					
Radius	SCP	95% CI	DCP	95% CI	CC	95% CI
<0.5 mm	11.9 ± 7.6	8.1–15.7	10.4 ± 6.3	7.3–13.5	14.2 ± 9.2	9.6–18.8
<0.75 mm	11 ± 6.5	7.8–14.2	10.7 ± 5.9	7.8–13.6	14.9 ± 9.5	10.2–19.6
<1.5 mm	12.7 ± 6.7	9.4–16	13.5 ± 7.9	9.6–17.4	17.6 ± 10.6	12.3–22.9

T_0_: baseline; T_6_: 6-month; SCP: superficial capillary plexus; DCP: deep capillary plexus; CC: choriocapillaris. CI95%: 95% confidence interval.

**Table 3:** Linear mixed model of the microvascular displacement in microns (µm) at different microvascular levels.

			95% CI	
Microvascular Slabs	Estimate	SE	Lower	Upper
Superficial	13.393	1.273	10.897	15.888
Deep	14.590	1.614	11.426	17.754
Choriocapillaris	18.517	1.182	16.200	20.834

CI: confidence interval.

## Data Availability

Data are available upon reasonable request directed to the corresponding author, Mariacristina Parravano, due to privacy restrictions.

## Abbreviations

The following abbreviations are used in this manuscript:

AMD	Age-related macular degeneration
CC	Choriocapillaris
DCP	Deep capillary plexus
dPED	Drusenoid pigment epithelial detachment
OCTA	Optical coherence tomography angiography
RPD	Reticular pseudodrusen
SDD	Subretinal drusenoid deposits
SCP	Superficial capillary plexus
